# Comparison of frozen-thaw blastocyst transfer strategies in women aged 35–40 years: a retrospective study

**DOI:** 10.3389/fendo.2023.1141605

**Published:** 2023-06-19

**Authors:** Yanhong Wu, Xiaosheng Lu, Haoying Chen, Yanghua Fu, Junzhao Zhao

**Affiliations:** Department of Reproductive Center, Obstetrics and Gynecology, the Second Affiliated Hospital and Yuying Children’s Hospital of Wenzhou Medical University, Wenzhou, China

**Keywords:** assisted reproductive technology (ART), frozen-thawed embryo transfer (FET), single blastocyst transfer (SBT), twin pregnancy, advanced maternal age (AMA)

## Abstract

**Objective:**

To compare the effects of five different frozen-thaw embryo transfer (FET) strategies in women aged 35–40 years.

**Methods:**

Data from 1,060 patients were divided into five groups according to the number and quality of transferred blastocysts: a high-quality single blastocyst group (group A, n= 303), a high-quality double blastocysts group (group B, n= 176), a high-quality plus poor-quality double blastocysts group (group C, n= 273), a poor-quality double blastocysts group (group D, n= 189), and a poor-quality single blastocyst group (group E, n= 119). Comparative analyses were then performed between groups with regard to primary conditions, pregnancy, and neonatal outcomes.

**Results:**

Group A had the lowest twin pregnancy rate (1.97%) and incidence of low-birth-weight infants (3.45%), which were significantly different from groups B, C, and D. In addition, the preterm birth rate (7.89%), neonatal birth weight (3300 g [3000, 3637.5]), and neonatal birth age (39.14 weeks [38.43, 39.61]) in group A were different from those in groups B and C. Double blastocyst transfer (DBT) was associated with a 20.558-fold (Risk Ratio [RR]=20.558, 95% confidence interval [CI], 6.628–63.763) and 3.091-fold (RR=3.091, 95% CI, 1.69–5.653) increased risk of twin pregnancy and preterm delivery in unadjusted analysis, respectively, when compared with single blastocyst transfer (SBT). In the adjusted analysis, we observed similar risk estimates (adjusted RR=26.501, 95% CI, 8.503–82.592; adjusted RR=3.586, 95% CI, 1.899–6.769).

**Conclusion:**

Although, high-quality SBT resulted in a lower live birth rate than high-quality DBT, it also significantly reduced the risk of adverse pregnancies, thus resulting in more benefits for both the mother and baby. Collectively, our data indicate that high-quality SBT remains the optimal FET strategy for women aged 35–40 years and warrants further clinical application.

## Introduction

Since October 2015, the rate of advanced maternal age (AMA) has increased from 8.5% to 13.5% with the full implementation of the two-child policy in China, with a growth rate of 58.8% (1). Furthermore, the implementation of the three-child policy in July 2021 will increase this rate further. Women of advanced age with reduced or lost fertility often require assisted reproductive technology (ART). In clinical practice, considering the effect of age on pregnancy rates, some physicians choose to transfer multiple embryos at once to achieve pregnancy as soon as possible in women of advanced age, thus making multiple pregnancies inevitable. Data from the Chinese Medical Association in 2016 showed that the twin pregnancy rate of frozen-thawed embryo transfer (FET) was 24.2% (2); this was much higher than in many other countries, including the United States (17.0%) (3) and Europe (10.1%) (4). Furthermore, there is a gap in the current internationally accepted control standard for the multiple pregnancy rate of ART (10%) (5). Pregnancy in AMA carries a high-risk of adverse pregnancy and the consequences will be exacerbated in the case of multiple pregnancy (6). Consequently, it is a serious challenge for reproductive physicians to help older infertile couples to achieve pregnancy while following the ethical principles of patient benefit and offspring protection.

Single embryo transfer (SET) is considered the best choice to reduce the rate of multiple pregnancies and improve perinatal outcomes (7). Blastocyst transfer is known to result in superior pregnancy outcomes when compared to the transfer of cleavage embryos (8). Currently, single blastocyst transfer (SBT) is generally used for young patients with a better prognosis; a previous study confirmed that frozen-thawed SBT is associated with a higher singleton live birth rate than fresh SBT (9). In our latest study, we found that high-quality SBT is the optimal FET strategy for young women aged < 35 years (10); however, the feasibility and efficacy of this strategy for women of advanced age has not been reported and needs to be confirmed by further studies. Therefore, in the present study, we conducted a comparative analysis of five different FET strategies to investigate the efficacy and feasibility of implementing a high-quality SBT protocol for women aged 35–40 years. Our aim was to provide a basis for an optimal FET transfer strategy for women of advanced age.

## Materials and methods

### Research objects

A retrospective analysis was performed on women who underwent FET at the Reproductive Center of the Second Affiliated Hospital of Wenzhou Medical University between January 2016 and August 2021. The inclusion criteria were as follows: (i) age 35–40 years; (ii) the thickness of the endometrial layer was greater than 7 mm on the day of endometrial transformation; (iii) no more than three transplantation cycles; (iv) no more than two day 5 (D5) blastocysts were transferred; and (v) the endometrial preparation was hormone replacement therapy (HRT). The exclusion criteria were as follows: (i) uteri with abnormal ultrasound findings such as endometrial polyps, endometrial fibroids, uterine adhesion, adenomyosis, or reproductive tract malformations; (ii) malignant tumors or other systemic chronic diseases, including those of the autoimmune system or hematological system; (iii) a history of genetic disorders in either of the couple receiving treatment; (iv) a history of habitual miscarriage or repeated implant failure; and (v) blastocysts undergoing preimplantation genetic testing (PGT).

**Figure 1:**
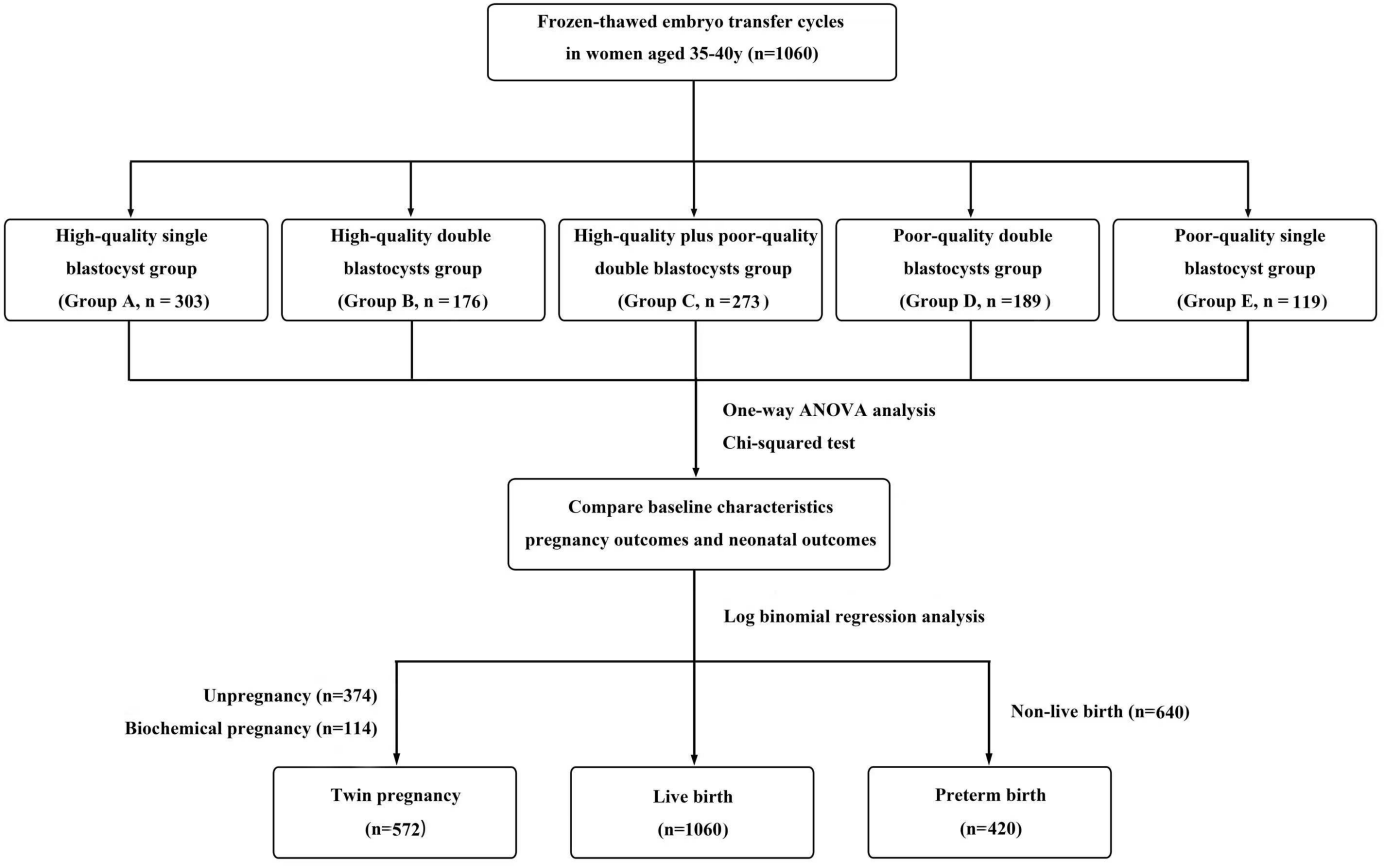
Flow chart. A total of 1,060 cycles received frozen-thawed embryo transfer (FET) in the reproductive center of the Second Affiliated Hospital of Wenzhou Medical University from January 2016 to August 2021. Among them, according to the number and quality of transferred blastocysts, the patients were divided into five groups: a high-quality single blastocyst group (group A, n=303), a high-quality double blastocysts group (group B, n=176), a high-quality plus poor-quality double blastocysts group (group C, n=273), a poor-quality double blastocysts group (group D, n=189), and a poor-quality single blastocyst group (group E, n=119). Statistical analysis was used to compare patients’ data.

A total of 1,060 eligible patients were enrolled in this study and divided into five groups according to the number and quality of transferred blastocysts: a high-quality single blastocyst group (group A, n = 303), a high-quality double blastocysts group (group B, n = 176), a high-quality plus poor-quality double blastocysts group (group C, n = 273), a poor-quality double blastocysts group (group D, n = 189), and a poor-quality single blastocyst group (group E, n = 119) (**Figure 1**). All methods used in this study were implemented in strict accordance with the consensus on human *in vitro* fertilization and embryo transfer (IVF-ET) laboratory manipulations (2016), the infertility diagnosis guide, and the Chinese expert consensus with regard to the number of embryos transferred.

### Hormone replacement therapy

**Figure 2:**
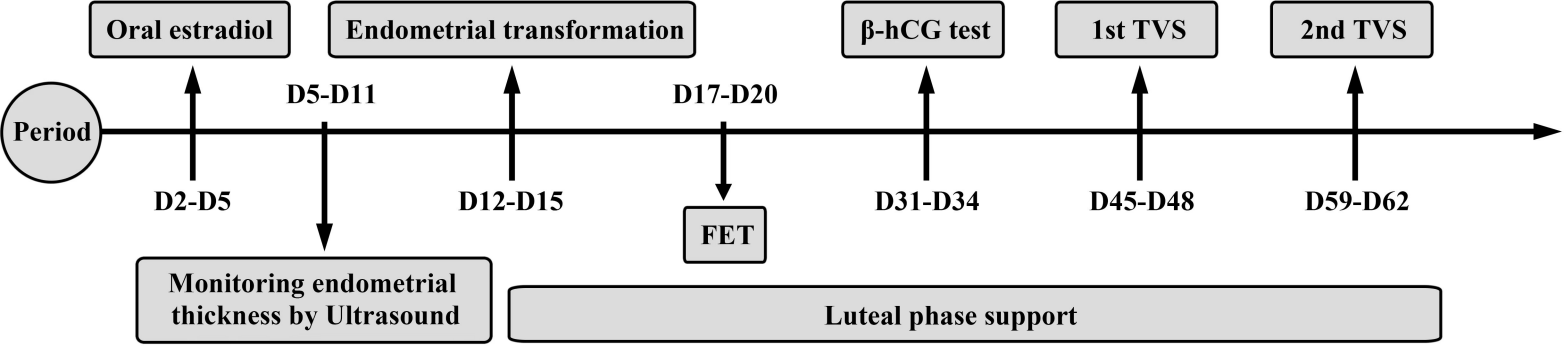
Hormone replacement therapy. Estradiol was taken from day 2 to day 5 of the menstrual cycle. Endometrial thickness was monitored by ultrasound every 3-5 days. When the endometrial thickness was greater than or equal to 7 mm and the progesterone level was less than 1.2ng/ml, progesterone would be given to start the endometrial transformation. Blastocyst transfer was performed on the 5th day after the endometrial transformation. The post-transplant luteal support regimen was the same as the post-transformation dosing. The first β-hCG test was performed 12–14 days after FET. The first ultrasound was performed 26–28 days after FET and the second ultrasound was performed 40–42 days after FET.

One tablet of estradiol (Femoston; Abbott Biologicals B.V.; Dose: 2 mg estradiol/tablet) was taken orally (twice a day) from day 2 to day 5 of the menstrual cycle. Endometrial thickness was monitored by ultrasound every 3–5 days and the dosage of estradiol tablets was adjusted according to the thickness of the endometrium. When the endometrial thickness was greater than or equal to 7 mm, and the level of progesterone was less than 1.2 ng/mL, we added 10 mg dydrogesterone tablets (Duphaston; Solvay Pharmaceuticals B.V.; Dose: 10 mg/tablet) twice a day for endometrial transformation, in addition to the one tablet of estradiol (orally twice a day). In addition, 200 mg of micronized progesterone was also administered orally or vaginally twice a day (Utrogestan; Capsugel, Besins Manufacturing Belgium, Bruxelles, Belgium; Dose: 0.1 g/tablet). Blastocyst transfer was performed on the 5^th^ day after endometrial transformation. The post-transplant luteal support regimen was the same as the post-transformation dosing (**Figure 2**).

### Thawing and culturing frozen-thawed embryos

Blastocyst thawing was performed on the morning of FET using a vitrification resuscitation kit (Vitrification VT102, Kitazato, Japan). After removing the cannulae from liquid nitrogen, the carrier rods were removed and quickly placed in thawing solution (TS) for 1 min at room temperature. Blastocysts were then transferred to dilution solution (DS) for 3 min, washing solution 1 (WS1) for 5 min, and washing solution 2 (WS2) for 5 min, respectively. Finally, blastocysts were transferred to blastocyst culture solution for observation and scoring before being placed in a three-gas incubator at 37°C, 6% CO_2_, and 5% O_2_ for 2 h before transfer.

### Criteria for blastocyst evaluation

The thawed blastocysts were graded according to Gardner’s grading criteria (11). In brief, blastocysts were divided into six stages according to the degree of blastocyst expansion and hatching, and were classified into A, B and C categories according to the development of the inner cell mass (ICM) and trophoblastic ectodermal cells (TE), respectively. Embryos with a development stage higher than 2 and both ICM and TE scores ≥ C were defined as poor-quality blastocysts, whereas those with ICM and TE scores ≥ B were defined as high-quality blastocysts.

### Criteria for determining pregnancy outcomes

The first β-hCG test was performed 12–14 days after FET. The first ultrasound was performed 26–28 days after FET and the second ultrasound was performed 40–42 days after FET (**Figure 2**). A biochemical pregnancy was defined as a β-hCG ≥15 mlU/mL on day 45 after FET and the absence of a gestational sac on ultrasound. Clinical pregnancy was defined as the presence of a gestational sac in the uterus with a heartbeat on ultrasound. Miscarriage was defined as the termination of pregnancy at less than 28 weeks of gestation with a fetus weighing less than 1 kg. Preterm delivery was defined as a delivery between 28 and 37 weeks of gestation. A newborn with a birth weight <2500 g was considered as a low-birth weight infant, while a newborn with a birth weight ≥ 4000 g was diagnosed with fetal macrosomia.

### Statistical methods

SPSS (version 26.0; IBM, Chicago) software was used for statistical analysis. Continuous variables that conformed to a normal distribution were expressed by mean and standard deviation or median, and comparisons between the five groups were performed using one-way analysis of variance (ANOVA). Measured variables that did not conform to a normal distribution were expressed by median and interquartile range (IQR), and differences between the five groups were compared using the Kruskal-Wallis H test. Differences in the measured variables between groups were analyzed by independent t-tests and Mann-Whitney U-tests. Count variables were compared using Pearson’s Chi-squared tests or Fisher’s exact tests. Log binomial regression analysis was used to analyze the main exposure factors affecting live birth, twin pregnancy, and preterm delivery, while unadjusted risk ratios (RRs), adjusted risk ratios (aRRs) and 95% confidence intervals (CI) were calculated for independent variables. *P*<0.05 was considered statistically significant.

## Results

There were no significant differences between the five groups in terms of maternal age, the duration of infertility, female BMI, maternal educational level, female ethnicity, the type and cause of infertility, or the endometrial thickness on the day of transformation (all *P*>0.05). The percentage of the first transplant cycles were statistically different in groups A and E when compared with the other three groups (50.50%, 44.54% vs. 29.55%, 27.47% and 27.51%, all *P*<0.001). The proportions of patients in the second transplant cycle in groups B, C, and D were significantly different from those in groups A and E (53.41%, 53.11%, and 56.61% vs. 34.65%, 38.66%, all *P*<0.001). However, the differences among the five groups in the third transplant cycle were not statistically significant (*P*>0.05; **Table 1**).

**Table 1:** Comparison of Baseline characteristics.

	Group A (n=303)	Group B (n=176)	Group C (n=273)	Group D (n=189)	Group E (n=119)	*P* value
Maternal age, mean(SD) (year)	37.47±1.62	37.22±1.70	37.37±1.65	37.41±1.66	37.53±1.68	0.477
Infertility duration, mean(SD)(year)	3.96±3.59	3.98±4.71	3.69±3.27	3.77±2.97	3.875±3.72	0.891
Female BMI, mean(SD)(kg/m^2^)	21.92±2.91	22.26±3.06	22.13±2.87	21.90±2.80	22.21±2.88	0.635
Maternal educational level						
Senior high school degree or below%(n)	57.10(173/303)	56.82(100/176)	58.24(159/273)	59.79(113/189	58.82(70/119)	0.975
Junior college or bachelor degree%(n)	34.98(106/303)	33.52(59/176)	31.14(85/273)	33.86(64/189)	34.46(41/119)	0.903
Master's degree or above%(n)	7.92(38/303)	9.66(17/176)	10.62(29/273)	6.35(12/189)	6.72(8/119)	0.158
Female ethnicity						
Han nationality%(n)	96.04(291/303)	96.02(169/176)	93.41(255/273)	94.18(178/189)	94.96(113/119)	0.608
Others%(n)	3.96(12/303)	3.98(7/176)	6.59(18/273)	5.82(11/189)	5.04(6/119)	0.608
Infertility type						
Primary infertility%(n)	11.88(36/303)	14.77(26/176)	15.38(42/273)	15.34(29/189)	16.81(20/119)	0.647
Secondary infertility%(n)	88.12(267/303)	85.23(150/176)	84.62(231/273)	84.66(160/189)	83.19(99/119)	0.647
Infertile causes						
Female factor%(n)	65.35(198/303)	70.45(124/176)	72.16(197/273)	71.43(135/189)	66.39(79/119)	0.374
Male factor%(n)	15.84(48/303)	13.64(24/176)	12.09(33/273)	11.64(22/189)	11.76(14/119)	0.599
Both factors%(n)^	9.57(29/303)	8.52(15/176)	8.42(23/273)	10.05(19/189)	11.76(14/119)	0.854
Unexplained factor%(n)	9.24(28/303)	7.39(13/176)	7.33(20/273)	6.88(13/189)	10.09(12/119)	0.759
Transplant cycle						
First cycle%(n)	50.50(153/303)^a,b,c^	29.55(52/176)	27.47(75/273)	27.51(52/189)	44.54(53/119)^g,i,j^	<0.001*
Second cycle%(n)	34.65(105/303)^a,b,c^	53.41(94/176)	53.11(145/273)	56.61(107/189)	38.66(46/119)^g,i,j^	<0.001*
Third cycle%(n)	14.85(45/303)	17.04(30/176)	19.42(53/273)	15.88(30/189)	16.80(20/119)	0.684
Endometrial thickness on the transformation day,mean(SD)(mm)	8.84±1.31	8.89±1.25	8.83±1.40	8.83±1.24	8.86±1.34	0.765

^^^Both factors were defined as more than one reason causing infertility.

^*^ *P* < 0.05 was statistical signifificance. “a” represents *P* value less than 0.05 between groups A and B, “b” represents *P* value less than 0.05 between groups A and C, “c” represents *P* value less than 0.05 between groups A and D, “d” represents *P* value less than 0.05 between groups A and E, “e” represents *P* value less than 0.05 between groups B and C, “f” represents *P* value less than 0.05 between groups B and D,”g” represents *P* value less than 0.05 between groups B and E,”h” represents *P* value less than 0.05 between groups C and D,”i” represents *P* value less than 0.05 between groups C and E,”j” represents *P* value less than 0.05 between groups D and E.

SD, Standard deviation; BMI, Body Mass Index.

There were no significant differences between the five groups with regard to the rate of biochemical pregnancy, rate of miscarriage, rate of ectopic pregnancy, the incidence of macrosomia, neonatal sex ratio, or the rate of obstetric complications (all *P*>0.05). The proportion of positive hCG tests (76.70% vs. 60.73%, 61.90%, 37.82%, respectively; all *P*<0.001) and the clinical pregnancy rate (69.32% vs. 50.17%, 48.68%, 31.09%, respectively; all *P*<0.001) were significantly higher in group B than in the other three groups, except for group C. The embryo implantation rate was significantly higher in group A (51.16%) than in groups C (39.93%; *P*=0.002), D (28.84%; *P*<0.001), and E (31.09%; *P*<0.001). The twin pregnancy rate (1.97% vs. 45.90%, 29.59%, 19.57%, respectively; all *P<*0.001) in group A was significantly different from that in groups B, C, and D. The preterm birth rate was significantly lower in group A (7.89%) than in groups B (33.70%; *P*<0.001) and C (23.33%; *P*=0.001). Group B (52.27%) had the highest live birth rate which was significantly different from that in groups A (37.62%; *P*=0.002), D (35.98%; *P*=0.002), and E (21.85%; *P*<0.001). In group A, the neonatal birth weight and neonatal birth age were significantly higher than in groups B and C. The incidence of low-birth weight infants was lowest in group A; this was significantly different from that in groups B, C, and D (3.45% vs. 30.40%, 25.83%, 22.08%, respectively; all *P<*0.001). The proportion of male infants born in group E was significantly lower than that in the other four groups (30.77% vs. 56.90%, 55.20%, 56.95%, 61.04%, respectively; all *P*<0.001; **Table 2**).

**Table 2:** Comparison of pregnancy outcomes and neonatal outcomes.

	Group A (n=303)	Group B (n=176)	Group C (n=273)	Group D (n=189)	Group E (n=119)	*P* value
hCG positive rate %(n)	60.73(184/303)^a,b^	76.70(135/176)	75.09(205/273)	61.90(117/189)^f,h^	37.82(45/119)^d,g,i,j^	<0.001^*^
Clinical pregnancy rate%(n)	50.17(152/303)^a,b^	69.32(122/176)	61.90(169/273)	48.68(92/189)^f,h^	31.09(37/119)^d,g,i,j^	<0.001^*^
Embryo implantation rate%(n)	51.16(155/303)^b,c^	50.57(178/352)^e^	39.93(218/546)	28.84(109/378)^f,h^	31.09(37/119)^d,g^	<0.001^*^
Biochemical pregnancy rate%(n)	10.56(32/303)	7.39(13/176)	13.19(36/273)	13.23(25/189)	6.72(8/119)	0.136
Miscarriage rate%(n)	24.34(37/152)	22.95(28/122)	28.99(49/169)	26.09(24/92)	29.73(11/37)	0.762
Ectopic pregnancy rate%(n)	0.66(1/152)	1.64(2/122)	0(0/169)	0(0/92)	0(0/37)	0.391
Twin pregnancy rate%(n)	1.97(3/152)^a,b,c^	45.90(56/122)^e^	29.59(50/169)^#^	19.57(18/92)^&,f^	0(0/47)^g,i,j^	<0.001^*^
Preterm birth rate%(n)	7.89(9/114)^a,b^	33.70(31/92)	23.33(28/120)	13.24(9/68)^f^	7.69(2/26)^g,i^	<0.001^*^
34 weeks ≤ gestational age <37 weeks	7.02(8/114)^a^	25.00(23/92)^e^	14.17(17/120)	8.82(6/68)^f^	7.69(2/26)^g^	0.004^*^
28 weeks ≤ gestational age <34 weeks	0.87(1/114)^a,b^	8.70(8/92)	9.17(11/120)	4.41(3/68)	0(0/26)	0.015^*^
Live birth rate%(n)	37.62(114/303)^a^	52.27(92/176)	43.96(120/273)	35.98(68/189)^f^	21.85(26/119)^d,g,i,j^	<0.001^*^
Neonatal birth weight, median(IQR) (g)^U^	3300(3000,3637.5)^a,b^	2740(2400,3400)	2900(2450,3430)	3100(2520,3600)	3275(3037.5,3762.5)^g,i^	<0.001^*^
Neonatal birth age, median(IQR)(weeks)^U^	39.14(38.43,39.61)^a,b^	38.22(36.43,39.26)	38.29(37.14,39.57)	38.79(37.33,39.43)	39.14(37.36,39.57)^g,i^	<0.001^*^
Incidence of macrosomia%(n)	5.17(6/116)	4.00(5/125)	5.96(9/151)	6.49(5/77)	3.85(1/26)	0.939
Incidence of low birth weight infants%(n)	3.45(4/116)^a,b,c^	30.40(38/125)	25.83(39/151)	22.08(17/77)	3.85(1/26)^g,i,j^	<0.001^*^
1500g≤birth weight<2500g	2.59(3/116)^a,b,c^	28.80(36/125)	22.52(34/151)	19.48(15/77)	3.85(1/26)^g,i^	<0.001^*^
Birth weight<1500g	0.86(1/116)	1.60(2/125)	3.31(5/151)	2.60(2/77)	0(0/26)	0.688
Neonatal sex ratio%(n)						
Male	56.90(66/116)	55.20(69/125)	56.95(86/151)	61.04(47/77)	30.77(8/26)^d,g,i,j^	0.107
Female	43.10(50/116)	44.80(56/125)	43.05(65/151)	38.96(30/77)	69.23(18/26)^d,g,i,j^	0.107
Obstetric complications						
Gestational hypertension%(n)	1.97(3/152)	0.82(1/122)	2.37(4/169)	1.09(1/92)	0(0/37)	0.881
ICP%(n)	0(0/152)	0.82(1/122)	0(0/169)	0(0/92)	0(0/37)	0.439
GDM%(n)	5.26(8/152)	3.28(4/122)	3.55(6/169)	1.09(1/92)	2.70(1/37)	0.577

^#^:In Group C, 50 multiple pregnancies included 1 monochorionic diamniotic twins, and the remaining 49 were dichorionic diamniotic twins.

^&^:In Group D, 18 multiple pregnancies included 1 monochorionic diamniotic twins, and the remaining 17 were dichorionic diamniotic twins.

^*^ *P* < 0.05 was statistical significance. “a” represents *P* value less than 0.05 between groups A and B, “b” represents *P* value less than 0.05 between groups A and C, “c” represents *P* value less than 0.05 between groups A and D, “d” represents *P* value less than 0.05 between groups A and E, “e” represents *P* value less than 0.05 between groups B and C, “f” represents *P* value less than 0.05 between groups B and D,”g” represents *P* value less than 0.05 between groups B and E,”h” represents *P* value less than 0.05 between groups C and D,”i” represents *P* value less than 0.05 between groups C and E,”j” represents *P* value less than 0.05 between groups D and E.

IQR, Interquartile range; ICP, Intrahepatic Cholestasis; GDM, Gestational Diabetes Mellitus.

^U^:Kruskal-Wallis H test/groups individually tested by Mann-Whitney U-test.

In the unadjusted analysis, an age >37 years was associated with a 0.802-fold increase in live birth outcomes when compared to 35–37 years (RR = 0.802; 95% CI: 0.691–0.931). In the adjusted analysis, we observed a similar risk estimate (aRR=0.809; 95% CI: 0.697–0.939). When stratified by the number of transplant cycles, women who received a third transplant had the lowest adjusted RR of 0.699 (95% CI: 0.545–0.897). The unadjusted RR for DBT versus SBT for live birth outcomes was 1.344 (95% CI: 1.143–1.597); the adjusted RR was 1.451 (95% CI: 1.216–1.732). Other factors associated with live birth outcomes in the unadjusted analysis included high-quality blastocyst transfer (RR=1.42; 95% CI: 1.178–1.713). In the adjusted analysis, women who transferred high-quality blastocysts had a 1.296-fold increase in live birth outcomes (aRR=1.296; 95% CI: 1.042–1.613) (**Table 3**).

**Table 3:** Unadjusted and adjusted analyses of live birth.

Factors	Crude		Adjusted	
	RR (95% CI)	*P* value	RR (95% CI)	*P* value
Infertility duration (year)				
<3	Ref		Ref	
≥3	0.952(0.821-1.105)	0.518	1.003(0.867-1.160)	0.965
BMI(kg/m^2^)				
<18.5	Ref		Ref	
18.5≤BMI<24	1.423(0.996-2.033)	0.053	1.416(0.996-2.013)	0.053
24≤BMI<28	1.419(0.967-2.083)	0.074	1.454(0.996-2.123)	0.053
≥28	1.576(0.961-2.583)	0.072	1.606(0.988-2.611)	0.056
Female age(year)				
35≤ age ≤37	Ref		Ref	
>37	0.802(0.691-0.931)	0.004	0.809(0.697-0.939)	0.005*
Infertility type				
Primary infertility	Ref		Ref	
Secondary infertiliy	0.993(0.804-1.225)	0.946	1.022(0.832-1.256)	0.834
Transplant cycle				
First cycle	Ref		Ref	
Second cycle	0.944(0.806-1.104)	0.469	0.916(0.783-1.07)	0.267
Third cycle	0.708(0.551-0.91)	0.007*	0.699(0.545-0.897)	0.005*
Number of blastocyst transfer				
Single	Ref		Ref	
Double	1.344(1.143-1.579)	<0.001*	1.451(1.216-1.732)	<0.001*
High-quality blastocyst transfer				
No	Ref		Ref	
Yes	1.42(1.178-1.713)	<0.001*	1.296(1.042-1.613)	0.02*
PCOS				
No	Ref		Ref	
Yes	0.931(0.729-1.189)	0.568	0.85(0.665-1.086)	0.193
Endometriosis				
No	Ref		Ref	
Yes	0.921(0.655-1.296)	0.638	0.88(0.631-1.227)	0.452
Endometrial thickness (mm)				
<8	Ref		Ref	
8-12	1.158(0.949-1.412)	0.149	1.076(0.886-1.307)	0.461
>12	1.184(0.713-1.966)	0.513	1.23(0.746-2.029)	0.416
Male factor				
No	Ref		Ref	
Yes	1.023(0.858-1.221)	0.797	1.036(0.874-1.227)	0.686
Maternal educational level				
Senior high school degree or below	Ref		Ref	
Junior college or bachelor degree	1.062(0.822-1.315)	0.762	1.012(0.701-1.213)	0.573
Master’s degree or above	0.981(0.718-1.243)	0.527	0.942(0.635-1.208)	0.632
Female ethnicity				
Han nationality	Ref		Ref	
Others	1.107(0.962-1.316)	0.621	1.095(0.874-1.227)	0.699

^*^ *P* < 0.05 was statistical significance.

BMI, Body Mass Index; PCOS, Polycystic Ovary Syndrome.

Several factors were associated with an increased risk of twin pregnancy in the unadjusted analysis. First, the risk of secondary infertility was lower than that of primary infertility (RR=0.659; 95% CI: 0.459–0.948). Second, DBT was associated with a higher risk compared to SBT (RR=20.558; 95% CI: 6.628–63.763). Third, the transfer of high-quality blastocysts had a higher risk of twin pregnancy than the transfer of poor-quality blastocysts (RR=1.763; 95% CI: 1.115–2.789. The number of blastocysts transferred, along with the type of blastocyst transfer, remained significant in the adjusted analysis. DBT was associated with a 26.501-fold increased risk of twin pregnancy (aRR=26.501; 95% CI: 8.503–82.592) while high-quality blastocyst transfer was associated with a 1.608-fold increased risk of twin pregnancy (aRR=1.608; 95% CI: 1.119–2.622) (**Table 4**).

**Table 4:** Unadjusted and adjusted analyses of twin pregnancy.

Factors	Crude		Adjusted	
	RR (95% CI)	*P* value	RR (95% CI)	*P* value
Infertility duration (year)				
<3	Ref		Ref	
≥3	1.255(0.92-1.712)	0.151	1.182(0.882-1.585)	0.264
BMI(kg/m^2^)				
<18.5	Ref		Ref	
18.5≤BMI<24	0.775(0.443-1.355)	0.371	0.863(0.527-1.414)	0.559
24≤BMI<28	0.702(0.369-1.333)	0.279	0.781(0.433-1.407)	0.411
≥28	1.409(0.677-2.932)	0.36	1.305(0.702-2.426)	0.400
Female age(year)				
35≤ age ≤37	Ref		Ref	
>37	0.808(0.592-1.102)	0.178	0.832(0.625-1.107)	0.208
Infertility type				
Primary infertility	Ref		Ref	
Secondary infertiliy	0.659(0.459-0.948)	0.024*	0.784(0.57-1.077)	0.132
Transplant cycle				
First cycle	Ref		Ref	
Second cycle	1.238(0.874-1.753)	0.229	0.86(0.629-1.177)	0.346
Third cycle	1.436(0.917-2.249)	0.114	0.961(0.641-1.441)	0.849
Number of blastocyst transfer				
Single	Ref		Ref	
Double	20.558 (6.628-63.763)	<0.001*	26.501(8.503-82.592)	<0.001*
High-quality blastocyst transfer				
No	Ref		Ref	
Yes	1.763(1.115-2.789)	0.015*	1.608(1.119-2.622)	0.009*
PCOS				
No	Ref		Ref	
Yes	1.396(0.924-2.109)	0.113	1.169(0.803-1.701)	0.416
Endometriosis				
No	Ref		Ref	
Yes	0.715(0.316-1.62)	0.422	0.723(0.327-1.6)	0.424
Endometrial thickness (mm)				
<8	Ref		Ref	
8-12	1.137(0.748-1.728)	0.548	1.077(0.741-1.564)	0.698
>12	1.071(0.366-3.135)	0.900	0.906(0.332-2.469)	0.847
Male factor				
No	Ref		Ref	
Yes	0.808(0.543-1.204)	0.295	0.856(0.599-1.222)	0.391
Maternal educational level				
Senior high school degree or below	Ref		Ref	
Junior college or bachelor degree	1.052 (0.825-1.612)	0.498	1.115(0.701-1.402)	0.321
Master’s degree or above	0.854 (0.654-1.342)	0.655	0.910(0.599-1.154)	0.463
Female ethnicity				
Han nationality	Ref		Ref	
Others	0.901 (0.511-1.720)	0.311	0.844(0.542-1.472)	0.632

^*^ *P* < 0.05 was statistical significance.

BMI, Body Mass Index; PCOS, Polycystic Ovary Syndrome.

In the unadjusted analysis, a BMI of 24–28 was associated with a 0.317-fold increased risk of preterm birth (RR=0.317; 95% CI: 0.11–0.909); however, in our adjusted analysis, we found that a BMI of 24–28 was not significantly associated with the risk of preterm birth. The unadjusted RR for the risk of preterm birth associated with DBT when compared with SBT was 3.091 (95% CI: 1.69–5.653) and the adjusted RR was 3.586 (95% CI: 1.899–6.769) (**Table 5**).

**Table 5:** Unadjusted and adjusted analyses of preterm birth.

Factors	Crude		Adjusted	
	RR (95% CI)	*P* value	RR (95% CI)	*P* value
Infertility duration (year)				
<3	Ref		Ref	
≥3	1.469(0.978-2.205)	0.064	1.412 (0.935-2.132)	0.101
BMI(kg/m^2^)				
<18.5	Ref		Ref	
18.5≤BMI<24	0.417(0.168-1.032)	0.059	0.709(0.374-1.344)	0.292
24≤BMI<28	0.317(0.11-0.909)	0.033*	0.541(0.247-1.183)	0.124
≥28	0.781(0.202-3.016)	0.72	0.986(0.384-2.532)	0.976
Female age(year)				
35≤ age ≤37	Ref		Ref	
>37	0.752(0.5-1.132)	0.173	0.809(0.527-1.242)	0.332
Infertility type				
Primary infertility	Ref		Ref	
Secondary infertility	0.669(0.415-1.078)	0.099	0.931(0.575-1.507)	0.77
Transplant cycle				
First cycle	Ref		Ref	
Second cycle	1.445(0.925-2.256)	0.106	1.18(0.759-1.834)	0.463
Third cycle	1.222(0.628-2.379)	0.555	0.942(0.491-1.81)	0.858
Number of blastocyst transfer				
Single	Ref		Ref	
Double	3.091(1.69-5.653)	<0.001*	3.586(1.899-6.769)	<0.001*
High-quality blastocyst transfer				
No	Ref		Ref	
Yes	1.782(0.984-3.229)	0.057	1.561(0.821-2.966)	0.174
PCOS				
No	Ref		Ref	
Yes	1.208(0.672-2.172)	0.528	0.931(0.517-1.676)	0.811
Endometriosis				
No	Ref		Ref	
Yes	1.222(0.551-2.714)	0.622	1.249(0.556-2.806)	0.59
Endometrial thickness (mm)				
<8	Ref		Ref	
8-12	1.024(0.607-1.727)	0.929	0.936(0.548-1.6)	0.809
>12	1.086(0.288-4.092)	0.903	0.772(0.199-2.992)	0.708
Male factor				
No	Ref		Ref	
Yes	0.791(0.473-1.323)	0.371	0.873(0.518-1.47)	0.609
Maternal educational level				
Senior high school degree or below	Ref		Ref	
Junior college or bachelor degree	1.143 (0.874-1.543)	0.412	1.211(0.831-1.596)	0.349
Master’s degree or above	1.039 (0.768-1.410)	0.701	0.981(0.602-1.268)	0.561
Female ethnicity				
Han nationality	Ref		Ref	
Others	0.946 (0.62-1.369)	0.742	1.061(0.813-1.212)	0.787

^*^ *P* < 0.05 was statistical significance.

BMI, Body Mass Index; PCOS, Polycystic Ovary Syndrome.

## Discussion

Despite the development of ART techniques, age remains an independent risk factor for fertility and pregnancy outcomes. Thus, women of advanced age have been the focus of ART research. Although one previous study showed that fresh blastocyst transfer is associated with a better pregnancy outcome than fresh cleavage transfer, and has gradually become the mainstream of embryo transfer at present (8), there is no clear evidence for how efficiently blastocyst transfer can be used in women of advanced age, especially in FET cycles. It remains a significant challenge for reproductive physicians to perform blastocyst transfer and achieve the best pregnancy outcomes for women of advanced age.

Several studies (12, 13) have confirmed that SBT in older infertile women can significantly reduce the rate of multiple pregnancies and the risk of adverse neonatal outcomes without reducing the live birth rate when compared with DBT; however, these studies only involved women aged 40–43 years and the transfer cycles were not all FET cycles. A previous meta-analysis (14) showed that high-quality SBT is feasible for women <40 years-of-age, but for women ≥40 years-of-age, the current evidence is insufficient to recommend an appropriate number of embryos for transfer. Very little evidence is available with regard to whether freeze-thaw SBT is appropriate for women of advanced age. In the present study, our analysis showed that group B yielded the highest hCG positive rate, clinical pregnancy rate, and live birth rate; these parameters were all significantly different from the other three groups (except for group C). This indicates that high-quality DBT in a FET cycle results in the best pregnancy outcomes. However, we should not overlook the fact that the high pregnancy rate associated with group B was accompanied by a significantly higher rate of twin pregnancy, preterm birth, and the incidence of low birth weight infants. In contrast, group A, with the transfer of one high-quality blastocyst, was associated with a lower live birth rate than group B (37.62% vs. 52.27%), but also a significant reduction in the rates of twin pregnancy, preterm birth, and low birth weight infants without increasing the incidence of adverse pregnancies. In addition, it is noteworthy that although the hCG positivity rate and clinical pregnancy rate were superior in group C compared to group A, the live birth rate (43.96% vs. 37.62%) did not differ significantly between the two groups. Furthermore, group C was associated with a significant increase in twin pregnancy rate, preterm birth rate, and low birth weight infants due to the transfer of double embryos. Log binomial regression analysis further revealed that DBT and high-quality blastocyst transfer were associated with a 1.451-fold and 1.296-fold increase in live birth outcome, respectively, thus confirming the significant advantage of high-quality DBT in terms of live birth rate. However, it should not be overlooked that DBT is also associated with an increased risk of twin pregnancy and preterm delivery (26.501-fold and 3.586-fold, respectively) when compared to SBT.

It is well known that preterm infants, especially low birth weight infants, have immature organ development and twin fetal pregnancies significantly increases the risk of perinatal diseases and complications in pregnant women, fetuses, and newborns. These factors also increase the risk of neonatal mortality and chronic diseases in adulthood, thus placing a heavy burden on the national health care system and the families involved (15). It has been argued that the high rate of multiple pregnancy and the additional complications associated with DBT outweigh the high live birth rate associated with DBT (16). The goal of ART is to achieve a full-term, singleton, healthy live birth, and the number of embryos transferred is a controllable factor that relates to the overall treatment outcome, maternal-fetal safety, and post-pregnancy health. Our analysis emphasizes that the risks of the entire pregnancy process must be considered when transferring embryos, rather than focusing only on clinical pregnancy rate and live birth rate.

Blastocyst quality is an important factor that can affect pregnancy outcomes. Previous researchers found that high-quality embryos may secrete microRNA hsa-miR-320a prior to implantation (17, 18). This miRNA signals the endometrium to induce the migration of human endometrial mesenchymal cells, thus providing a mechanism to continuously rebalance endometrial tolerance and selectivity characteristics, thereby making the underlying metaphase layer more receptive to invasion, thereby increasing the likelihood of successful pregnancy. However, poor-quality embryos are unable to secrete microRNA hsa-miR-320a; thus, this inactivates the network supporting the metaphase, and reducing the chances of embryo implantation (17, 18). In the present study, although the live birth rate in group C did not differ significantly from that in group A, the embryo implantation rate was significantly lower (39.93% vs. 51.16%), thus reducing the utilization of embryos. However, some researchers have argued that poor-quality embryos do not negatively affect the implantation potential of co-transferred high-quality embryos, but it is important to note that this comes at the cost of a significantly increased likelihood of twin pregnancy (19, 20). Thus, there is no conclusive evidence relating to whether poor-quality embryos affect embryo implantation, but it is certain that both strategies significantly increase the occurrence of twin pregnancy, whether ART treatment involves high-quality DBT or the simultaneous transfer of a high-quality blastocyst and a poor-quality blastocyst; thus, we do not promote the use of these techniques. On balance, with a surplus of blastocysts to choose from, we believe that high-quality SBT should be the preferred strategy for women aged 35–40 years in a FET cycle; this finding is consistent with a previous report by Chen et al. (21). It should be noted that there are some infertile women who cannot undergo this transfer strategy due to a low number of embryos and poor embryo quality; for this group of patients, the transfer option can only be selected according to the actual clinical situation.

Interestingly, in our previous study of young women aged < 35 years, we found that the proportion of male offspring increased significantly only with high-quality SBT and high-quality DBT (10). Furthermore, several studies (22, 23) have also concluded that high-quality blastocysts are more likely to result in male babies when compared to poor-quality blastocysts. In the present study, we found that the proportion of male offspring increased significantly in remaining four groups, except for group E. We hypothesize that the reason for this is that performing DBT in women of advanced age can compensate for the significant reduction in live birth rate, although the existence of competition in the early stage of embryo implantation may be more pronounced than in younger women. Furthermore, embryos of differing quality may have an impact on embryo implantation, thus leading to differences in the gender of the offspring. Only a few studies have investigated the sex of offspring born after ART over recent years, probably because the gender issue is relatively sensitive in China, and it is difficult to involve multiple studies with large sample numbers. Although at this stage, the proportion of post-ART offspring in the overall population is low and will not affect the sex ratio of the population in the short term, it is unclear how the development of ART, and the increasing population of newborns born through this technique, will affect the population composition in the future. This is one of the major issues that reproductive practitioners need to be aware of in the future.

There are three major limitations in this study that need to be considered. First, the study objects were not stratified by age, and only the effect of women aged 35–40 years on the primary outcome was considered. There is a significant lack of strong evidence as to whether this FET strategy is applicable to the women aged >40 years. It is hoped that this limitation will be addressed in future studies when more cases are collected. Second, we only investigated pregnancy outcomes after FET and did not analyze cumulative pregnancy rates or track the mental and physical development of newborns after birth. Finally, this study was a retrospective study carried out in a single center; there was no randomization of results. Thus, our results cannot be generalized; a large sample, prospective, multicenter, randomized, and controlled trial is now needed to validate the advantages of this FET strategy.

## Conclusions

It is vital that we fully investigate the range of maternal and infant safety issues associated with AMA while following the principles of patient benefit and offspring protection. In FET cycles, the choice of high-quality SBT for women aged 35–40 years results in both a high implantation rate and a significant reduction in the rates of twin pregnancy, preterm birth, and low birth weight infants, with more maternal and infant benefits. Although the live birth rate is not as high as that for high-quality DBT, on balance, it is still considered that high-quality SBT is the best FET strategy for women aged 35–40 years; this strategy should be implemented on a wider basis. The findings of this study have significant clinical implications for blastocyst selection strategies and the effective reduction of twin pregnancy rate in women of advanced age undergoing FET cycles.

## Data availability statement

The raw data supporting the conclusions of this article will be made available by the authors, without undue reservation.

## Ethics statement

The studies involving human participants were reviewed and approved by the Ethics Committee (Institutional Review Board) of the Second Affiliated Hospital and Yuying Children’s Hospital of Wenzhou Medical University (2022-K-191-01). The patients/participants provided their written informed consent to participate in this study. Written informed consent was obtained from the individual(s) for the publication of any potentially identifiable images or data included in this article.

## Author contributions

YW contributed to the conception and design of this study. XL acquired and interpreted the data. HC and YF wrote the first draft of the manuscript. JZ approved the final manuscript. All authors contributed to the article and approved the submitted version.
